# Polymyxins and Doripenem Combination Against KPC-Producing *Klebsiella pneumoniae*

**DOI:** 10.4021/jocmr1220w

**Published:** 2013-02-25

**Authors:** Grace C. Lee, David S. Burgess

**Affiliations:** a University of Texas Health Science Center at San Antonio, Pharmacotherapy Education and Research Center, 7703 Floyd Curl Drive - MC 6220, San Antonio, TX, U.S; b College of Pharmacy, University of Texas at Austin, 1 University Station, Austin, TX U.S; c Department of Pharmacy Practice and Science, University of Kentucky College of Pharmacy, 789 S. Limestone, 292K, Lexington, KY 40536, U.S

**Keywords:** *Klebsiella pneumoniae*, KPC, Carbapenemase, Time-kill, Doripenem, Colistin, Polymyxin B, Carbapenem, Synergy

## Abstract

**Background:**

Most KPC-producing organisms have maintained susceptibility to polymyxins; however, development of resistance to polymyxins has been increasingly reported. One potential treatment modality is to optimize the use of combination therapy. Therefore, we evaluated the in vitro activity of doripenem, colistin sulfate, polymyxin B alone and in combination against KPC- producing *K. pneumoniae*.

**Methods:**

In-vitro time-kill assays were performed for four non-duplicate KPC-3 producing *K. pneumoniae* isolates with the following antibiotics: doripenem, polymyxin B and colistin sulfate alone and in combination. Bacterial densities were determined at 0, 4, 8, 12, 24 and 48 hours. Bactericidal activity was defined as ≥ 3-log_10_ CFU/mL reduction from the starting inoculum. Synergism was defined as ≥ 2-log_10_ reduction with the combination when compared to the most active single agent at 24 hours.

**Results:**

Minimum inhibitory concentrations (MICs) for polymyxin B and colistin sulfate ranged from 0.0625 to 0.25 µg/mL, and all isolates were resistant to doripenem (MICs ranged 16 - 32 µg/mL). Monotherapy with colistin sulfate and polymyxin B displayed bacterialcidal activity within 12 hours; however, significant re-growth occurred by 24 hours in all isolates. Monotherapy with doripenem did not show bactericidal activity in any isolate. Synergy occurred with combinations of both colistin sulfate and polymyxin B with doripenem against all isolates and was sustained at 48 hours. Combinations of colistin sulfate or polymyxin B with doripenem demonstrated rapid bactericidal activity by 4 hours in all isolates and was sustained for 24 hours.

**Conclusion:**

Polymyxin B and colistin sulfate in combination with doripenem may be an important treatment modality in treating KPC-producing organisms.

## Introduction

Over the last decade, the detection of *Klebsiella pneumoniae* carbapenemase (KPC)-producing organisms have been increasing at an alarming rate in the United States and throughout the world [1]. Since its first report in 2001, 10 additional KPC variants have been subsequently discovered, with KPC-2 and KPC-3 as the most commonly reported [2, 3]. KPC enzymes have the capacity to confer resistance to all β-lactams including carbapenems. In addition, organisms harboring the *bla*_kpc_ gene are frequently resistant to other antibiotics commonly used against *Enterobacteriaceae* infections, further limiting therapeutic options [4]. As a result of their multi-drug resistant profiles, infections caused by KPC-producing organisms have been associated with increased treatment failures, longer hospital stays and high mortality rates [5-8].

Despite the rapid rise of *Klebsiella pneumoniae* carbapenemase (KPC) producing organisms, the optimal treatment for these infections has not been well established. KPC-producing organisms have historically maintained susceptibility to polymyxins (for example, colistin sulfate and polymyxin B). Unfortunately, polymyxin resistance can develop rapidly when used as monotherapy and incidences of polymyxin resistance are being reported with increasing frequency [9-11]. Several in vitro studies have demonstrated that while polymyxins can exhibit rapid killing against KPC-producing organisms, re-growth has been observed by 24 hours [12, 13]. In the era of increasing resistance coupled with a decreasing antibiotic armamentarium, attention must be brought to the role of combination therapy. We evaluated the in vitro activity of colistin sulfate, polymyxin B, doripenem alone and in combination against KPC- producing *K. pneumoniae*.

## Methods

Four non-duplicate KPC-3-producing *K. pneumoniae* isolates were collected from hospitalized patients previously described in an observational clinical case series [14]. Minimum inhibitory concentrations (MICs) were determined for doripenem, colistin sulfate, and polymyxin B by broth microdilution according to the Clinical and Laboratory Standards Institute (CLSI).

We evaluated the activity of the following regimens by conducting time-kill assays: doripenem, colistin sulfate, polymyxin B, colistin sulfate plus doripenem, and polymyxin B plus doripenem. Concentrations representative of achievable serum levels were used: doripenem concentration of 6 μg/mL and 2× MIC for colistin sulfate and polymyxin B. The methodology used for time-kill assays were previously described [15]. Bacterial densities were determined at 0, 4, 8, 12, 24 and 48 hours post inoculation. Fifty μLs were plated on trypticase soy agar plates using a spiral plater (Spiral Biotech, Bethesda, MD) then incubated for 24 hours before colony counts were enumerated using a laser colony counter (Q-Counter, Spiral Biotech, Bethesda, MD). Bactericidal activity was defined as ≥ 3-log_10_ CFU/mL reduction from the starting inoculum. Synergism and antagonism were defined as ≥ 2-log_10_ reduction or increase, respectively, in combination when compared to the most active single agent at 24 hours. The limit of quantification was 10^2^ CFU/mL. All tests were performed in duplicate. Isolates exposed to colistin sulfate or polymyxin B with viable colonies after 24 hours were subjected to susceptibility testing to evaluate the development of resistance. Development of resistance was defined by an MIC increase of > 4 times the initial MIC.

## Results

All strains were resistant to doripenem (MICs: 16 - 32 μg/mL) and susceptible to colistin sulfate and polymyxin B (Table 1).

**Table 1:** Minimum Inhibitory Concentrations (μg/mL) for KPC-3 Producing *Klebsiella pneumoniae*

	Isolates			
	C16	C17	C33	C50
**Doripenem**	32	32	16	16
**Colistin sulfate**	0.25	0.125	0.25	0.0625
**Polymyxin B**	0.25	0.125	0.25	0.125

**Figure 1:**
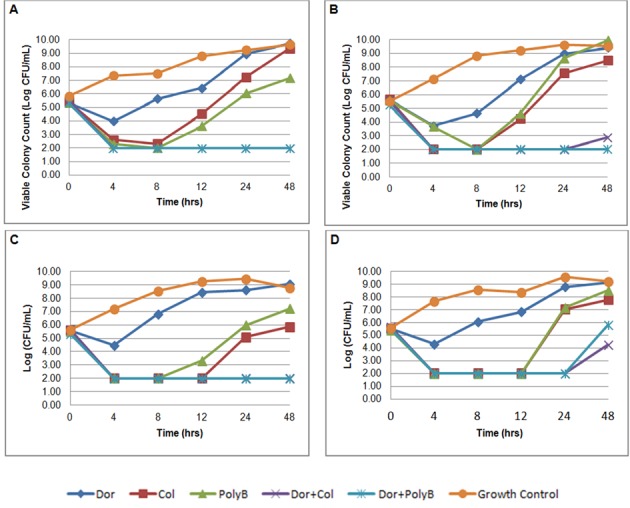
In-vitro time-kill assays of doripenem, colistin sulfate, polymyxin B alone and in combination against four KPC-3-producing *Klebsiella pneumoniae*. A) isolate C16; B) isolate C17; C) isolate C33; D) isolate C50.

The average bacterial density of the starting inoculum was 5.52 ± 0.12 log_10_. For doripenem monotherapy, an initial reduction in log CFU/mL was observed at 4 hours followed by regrowth at 8 hours for all isolates (Fig. 1). Doripenem alone did not achieve bactericidal activity in any isolate at any time point. Colistin sulfate alone exhibited rapid bactericidal activity in 3 of 4 (75%) isolates by 4 hours and in all (4/4) isolates by 8 hours. Significant regrowth occurred in all 4 isolates exposed to colistin sulfate monotherapy with an average colony count of 6.72 ± 1.11 log_10_ at 24 hours and to growth-control quantities by 48 hours. Likewise, bactericidal activity was not sustained after 8 hours for polymyxin B alone. At 24 hours, there were significant regrowth among all of the strains exposed to polymyxin B monotherapy with an average colony count of 6.97 ± 1.25 log_10_.

Both combinations of colistin sulfate plus doripenem and polymyxin B plus doripenem rapidly achieved bactericidal activity by 4 hours and were maintained through 24 hours in all strains. Synergy was observed for colistin sulfate plus doripenem in all isolates. Similarly, polymyxin B in combination with doripenem was synergistic and bactericidal at 24 hours. At 48 hours, the combination of colistin sulfate and doripenem demonstrated synergy against all 4 isolates and was bactericidal against 2 (50%) of the 4 isolates (C16 and C33). The combination of polymyxin B and doripenem demonstrated synergy against all 4 isolates at 48 hours and remained bactericidal in 3 (75%) of 4 isolates (C16, C17, and C33). Antagonism was not identified in any combination. Repeat MICs were performed for all 4 isolates with viable colonies after 24 hours of exposure to colistin sulfate and polymyxin B. All isolates exposed to colistin sulfate and polymyxin B alone developed resistance (MICs: 8 - 128 μg/mL) after 24 hours. Exposure to polymyxins had no change on doripenem MICs. Cross resistance between colistin sulfate and polymyxin B was also observed. After 24 hours of colistin sulfate exposure, the MIC for polymyxin B increased greater than about 500 fold. Colistin sulfate MICs increased about 32 - 2,000 fold post polymyxin B exposure.

## Discussion

The incidence of infections caused by KPC-producing organisms is increasing at an alarming rate. These pathogens are highly resistant to multiple antimicrobial classes. While KPC-producing bacteria have historically maintained susceptibility to polymyxins, resistance to these last line agents is now well-documented. Studies evaluating the role of combination therapies against these pathogens are urgently needed. Herein, we sought to evaluate the effectiveness of colistin sulfate and polymyxin B in combination with doripenem. To our knowledge, this is the first study to compare activities of both colistin sulfate and polymyxin B in combination with doripenem against KPC isolates.

Overall, our study demonstrated that while polymyxin B and colistin sulfate exhibited rapid bactericidal activity within 8 hours, regrowth occurred by 24 hours. None of the monotherapy regimens sustained bactericidal killing at 24 hours. In comparison, with the addition of doripenem to colistin sulfate and polymyxin B, bactericidal killing was achieved and sustained through 24 hours in all isolates. Moreover, an important clinical observation was the bactericidal and synergistic activity of these combinations using concentrations of polymyxins (0.12 - 0.5 μg/mL) lower than traditionally studied. Implications of these preliminary findings suggest that when used in combination with doripenem, aggressive dosing of polymyxins to achieve higher serum concentrations may not be necessary. Toward this end, the risk of polymyxin dose-limiting toxicities including nephrotoxicity and neurotoxicity may be minimized. It is unknown, however, if higher concentrations of polymyxins may have maintained bactericidal killing in combination in all 4 isolates at 48 hours.

### Conclusion

Polymyxins in combination with doripenem provided synergistic effects and achieved bactericidal activity minimizing the development of polymyxin resistance. Clinical studies and further in vitro studies evaluating a larger number of isolates and wider range of MICs are necessary to corroborate our findings.
